# (*E*)-*N*′-[1-(2-Hy­droxy­phen­yl)ethyl­idene]-2-phen­oxy­acetohydrazide–2,2′-(1,1′-azinodiethyl­idyne)diphenol (2/1)

**DOI:** 10.1107/S1600536811023956

**Published:** 2011-06-25

**Authors:** Yan-Ru Tang

**Affiliations:** a Department of Chemistry, Changchun Normal University, Changchun 130032, People’s Republic of China

## Abstract

The formula unit of the title mol­ecular complex, 2C_16_H_16_N_2_O_3_·C_16_H_16_N_2_O_2_, consists of two (*E*)-*N*′-[1-(2-hy­droxy­phen­yl)ethyl­idene]-2-phen­oxy­acetohydrazide mol­ecules and one mol­ecule of 2,2′-(1,1′-azinodiethyl­idyne)diphenol, with the latter located on a crystallographic inversion center. The acetohydrazide mol­ecules are linked into a supermolecular chain along the *c* axis by inter­molecular N—H⋯O hydrogen bonds. There are also intra­molecular O—H⋯N hydrogen bonds in both the acetohydrazide and diphenol mol­ecules.

## Related literature

For chemically related applications arising from Schiff base compounds, see: Guo *et al.* (2010 ▶); Yu *et al.* (2010 ▶). For related structures, see: Lu *et al.* (1993 ▶); Matoga *et al.* (2007 ▶); Tai *et al.* (2008 ▶); Tan (2009 ▶); Wen *et al.* (2005 ▶).
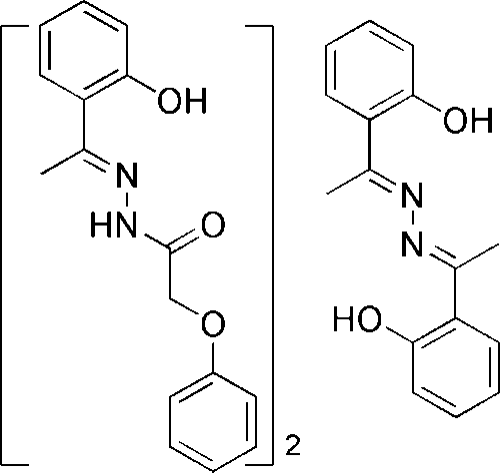


## Experimental

### 

#### Crystal data

2C_16_H_16_N_2_O_3_·C_16_H_16_N_2_O_2_
*M* *_r_* = 836.92
Monoclinic, 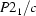

*a* = 12.416 (5) Å
*b* = 19.322 (6) Å
*c* = 9.225 (4) Å
β = 106.156 (16)°
*V* = 2125.8 (15) Å^3^
*Z* = 2
Mo *K*α radiation
μ = 0.09 mm^−1^
*T* = 293 K
0.18 × 0.15 × 0.13 mm

#### Data collection

Bruker APEXII CCD area-detector diffractometer
Absorption correction: multi-scan (*SADABS*; Bruker, 2004 ▶) *T*_min_ = 0.984, *T*_max_ = 0.988
20592 measured reflections
4828 independent reflections
2586 reflections with *I* > 2σ(*I*)
*R*_int_ = 0.072

#### Refinement

*R*[*F* ^2^ > 2σ(*F* ^2^)] = 0.068
*wR*(*F* ^2^) = 0.156
*S* = 1.03
4828 reflections
284 parameters
H-atom parameters constrained
Δρ_max_ = 0.16 e Å^−3^
Δρ_min_ = −0.19 e Å^−3^

### 

Data collection: *APEX2* (Bruker, 2004 ▶); cell refinement: *SAINT* (Bruker, 2004 ▶); data reduction: *SAINT*; program(s) used to solve structure: *SHELXS97* (Sheldrick, 2008 ▶); program(s) used to refine structure: *SHELXL97* (Sheldrick, 2008 ▶); molecular graphics: *DIAMOND* (Brandenburg, 1999 ▶); software used to prepare material for publication: *SHELXL97*.

## Supplementary Material

Crystal structure: contains datablock(s) I, global. DOI: 10.1107/S1600536811023956/ld2015sup1.cif

Structure factors: contains datablock(s) I. DOI: 10.1107/S1600536811023956/ld2015Isup2.hkl

Additional supplementary materials:  crystallographic information; 3D view; checkCIF report

## Figures and Tables

**Table 1:** Hydrogen-bond geometry (Å, °)

*D*—H⋯*A*	*D*—H	H⋯*A*	*D*⋯*A*	*D*—H⋯*A*
N2—H2*A*⋯O2^i^	0.86	2.14	2.860 (3)	141
O1—H01*A*⋯N1	0.96	1.63	2.530 (3)	154
O4—H04*A*⋯N3	1.06	1.58	2.542 (3)	148

Symmetry code: (i) 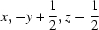.

**Fig. 1.**
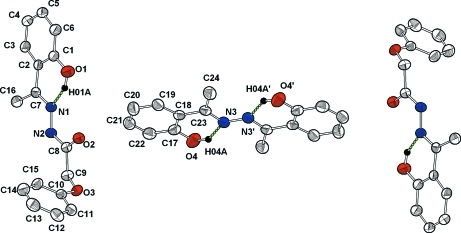
A view of the title organic compound, showing the atomic numbering scheme. Displacement ellipsoids are drawn at the 30% probability level.

**Fig. 2.**
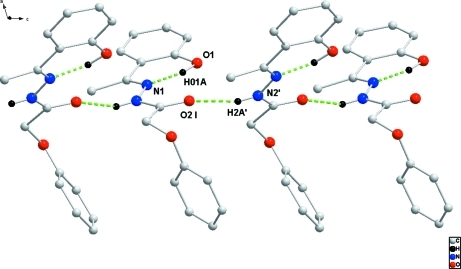
One-dimensional chain structure of molecules A. Hydrogen bonds are shown as green dashed lines [symmetry code: (I) x, 1/2 - y, -1/2 + z].

**Fig. 3.**
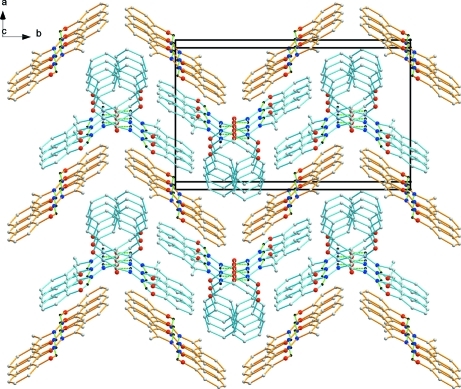
Packing diagram of molecules A (blue bonds) and B (orange bands).

## supplementary crystallographic information

### Comment

Among the richness of coordination chemistry, acylhydrazone ligands (Yu *et al.*, 2010) have attracted an intense interest due to their potential for magnetochemistry (Guo *et al.*, 2010). Recently, a large number of acylhydrazone derivatives have been prepared (Matoga *et al.*, 2007; Tan, 2009). As a contribution to this field, the isolation and the structure of the title 2/1 co-crystal are presented here .

The molecular structure of 2C_16_H_16_N_2_O_3_.C_16_H_16_N_2_O_2_, together with the atom-numbering scheme, is illustrated in Fig.1. Selected bond lengths and angles are given in Table 1. The asymmetric unit of the title co-crystal comprises two (*E*)-N'-(1-(2-hydroxyphenyl)ethylidene)-2-phenoxyacetohydrazide (A) molecules and a molecule of 2,2'-(1,1'-Azinodiethylidyne)diphenol (B), with no proton transfer.  The 2,2'-(1,1'-Azinodiethylidyne)diphenol molecule (B) has been reported previously (Tai *et al.*, 2008). The N3—N3A (1.394 (4) Å) distance is similar to the corresponding distances observed for other compounds (Lu *et al.*, 1993). In the molecule A, the N1—N2(hydrazine) bond distance of 1.375 (2) Å is shorter than the corresponding N—N value of 1.382 (2) Å in a related compound (Wen *et al.*, 2005). The dihedral angle between both aromatic rings for molecule A is 85.76 (2)°, and the molecules A are linked into supermolecule chain along the *c* axis by intermolecular N2—H2A···O2I hydrogen bonds [symmetry code: (I) *x*, 1/2 - *y*, -1/2 + *z*] (Fig. 2). In addition, there are intramolecular O1—H01A···N1 and O4—H04A···N3 hydrogen bonds in molecules A and B respectively. Stacking interactions between A and B are within van der Waals contacts (Fig.3).

### Experimental

A solution of 2,2'-(1,1'-azinodiethylidyne)diphenol (0.2 mmol) in 10 ml of EtOH was added to a solution of (*E*)-N'-(1-(2-hydroxyphenyl)ethylidene) -2-phenoxyacetohydrazide (0.2 mmol) in 10 ml of the same solvent, upon which the solution was refluxed for 1 h. Then the yellow solution was obtained after filtering. Two week later, yellow crystals of the title compound were isolated from the solution.

### Refinement

In the title compound, H atoms bonded to C/N atoms were positioned geometrically and refined using a riding and rotating (AFIX 137 for methyl hydrogens) model, with C—H = 0.93—0.97 Å, N—H = 0.86 Å, and *U*_ĩso_(H) = 1.2/1.5 *U*eq(C), *U*_ĩso_(H) = 1.2 *U*eq(N). H atoms bonded to phenolic OH groups were located from difference Fourier series and then allowed to ride on their parent O atoms (AFIX 3) with *U*_ĩso_(H) refined.

### Crystal data

**Table:**

C_48_H_48_N_6_O_8_	*F*(000) = 884
*M**_r_* = 836.92	*D*_x_ = 1.308 Mg m^−^^3^
Monoclinic, *P*2_1_/*c*	Mo *K*α radiation, λ = 0.71073 Å
Hall symbol: -P 2ybc	Cell parameters from 20942 reflections
*a* = 12.416 (5) Å	θ = 3.1–27.5°
*b* = 19.322 (6) Å	µ = 0.09 mm^−^^1^
*c* = 9.225 (4) Å	*T* = 293 K
β = 106.156 (16)°	Block, yellow
*V* = 2125.8 (15) Å^3^	0.18 × 0.15 × 0.13 mm
*Z* = 2	

### Data collection

**Table:**

Bruker APEXII CCD area-detector diffractometer	4828 independent reflections
Radiation source: fine-focus sealed tube	2586 reflections with *I* > 2σ(*I*)
graphite	*R*_int_ = 0.072
φ and ω scans	θ_max_ = 27.5°, θ_min_ = 3.1°
Absorption correction: multi-scan (*SADABS*; Bruker, 2004)	*h* = −16→15
*T*_min_ = 0.984, *T*_max_ = 0.988	*k* = −24→22
20592 measured reflections	*l* = −11→11

### Refinement

**Table:**

Refinement on *F*^2^	Primary atom site location: structure-invariant direct methods
Least-squares matrix: full	Secondary atom site location: difference Fourier map
*R*[*F*^2^ > 2σ(*F*^2^)] = 0.068	Hydrogen site location: inferred from neighbouring sites
*wR*(*F*^2^) = 0.156	H-atom parameters constrained
*S* = 1.03	*w* = 1/[σ^2^(*F*_o_^2^) + (0.0615*P*)^2^ + 0.318*P*] where *P* = (*F*_o_^2^ + 2*F*_c_^2^)/3
4828 reflections	(Δ/σ)_max_ < 0.001
284 parameters	Δρ_max_ = 0.16 e Å^−^^3^
0 restraints	Δρ_min_ = −0.19 e Å^−^^3^

### Special details

**Table:**

Geometry. All esds (except the esd in the dihedral angle between two l.s. planes) are estimated using the full covariance matrix. The cell esds are taken into account individually in the estimation of esds in distances, angles and torsion angles; correlations between esds in cell parameters are only used when they are defined by crystal symmetry. An approximate (isotropic) treatment of cell esds is used for estimating esds involving l.s. planes.
Refinement. Refinement of *F*^2^ against ALL reflections. The weighted R-factor wR and goodness of fit *S* are based on *F*^2^, conventional *R*-factors *R* are based on *F*, with *F* set to zero for negative *F*^2^. The threshold expression of *F*^2^ > 2sigma(*F*^2^) is used only for calculating R-factors(gt) etc. and is not relevant to the choice of reflections for refinement. *R*-factors based on *F*^2^ are statistically about twice as large as those based on *F*, and *R*- factors based on ALL data will be even larger.

### Fractional atomic coordinates and isotropic or equivalent isotropic displacement parameters (Å^2^)

**Table:**

	*x*	*y*	*z*	*U*_iso_*/*U*_eq_	
C1	0.6516 (2)	0.45868 (12)	0.6050 (3)	0.0530 (6)	
C2	0.62855 (18)	0.46636 (10)	0.4481 (3)	0.0436 (5)	
C3	0.6718 (2)	0.52452 (12)	0.3947 (3)	0.0559 (6)	
H3A	0.6575	0.5308	0.2911	0.067*	
C4	0.7351 (2)	0.57283 (12)	0.4910 (3)	0.0676 (8)	
H4A	0.7640	0.6109	0.4527	0.081*	
C5	0.7553 (2)	0.56461 (13)	0.6431 (3)	0.0714 (8)	
H5A	0.7975	0.5975	0.7084	0.086*	
C6	0.7140 (2)	0.50842 (13)	0.7003 (3)	0.0702 (8)	
H6A	0.7280	0.5036	0.8042	0.084*	
C7	0.56243 (18)	0.41504 (11)	0.3420 (2)	0.0434 (5)	
C8	0.45215 (18)	0.25229 (11)	0.3911 (3)	0.0435 (5)	
C9	0.3965 (2)	0.19347 (11)	0.2898 (3)	0.0528 (6)	
H9A	0.3438	0.2122	0.2002	0.063*	
H9B	0.4529	0.1676	0.2581	0.063*	
C10	0.24130 (19)	0.17197 (11)	0.3878 (2)	0.0452 (6)	
C11	0.1875 (2)	0.12496 (13)	0.4551 (3)	0.0589 (7)	
H11A	0.2182	0.0812	0.4810	0.071*	
C12	0.0881 (2)	0.14261 (17)	0.4840 (3)	0.0728 (8)	
H12A	0.0520	0.1108	0.5300	0.087*	
C13	0.0425 (2)	0.20653 (18)	0.4457 (4)	0.0790 (9)	
H13A	−0.0246	0.2185	0.4656	0.095*	
C14	0.0958 (2)	0.25275 (15)	0.3778 (4)	0.0799 (9)	
H14A	0.0646	0.2963	0.3512	0.096*	
C15	0.1955 (2)	0.23583 (13)	0.3482 (3)	0.0649 (7)	
H15A	0.2312	0.2676	0.3016	0.078*	
C16	0.5283 (2)	0.42865 (13)	0.1766 (3)	0.0602 (7)	
H16A	0.4707	0.3965	0.1273	0.090*	
H16B	0.5919	0.4232	0.1378	0.090*	
H16C	0.5001	0.4750	0.1580	0.090*	
C17	0.8581 (2)	0.08891 (13)	0.6727 (3)	0.0625 (7)	
C18	0.86204 (19)	0.11226 (12)	0.8180 (3)	0.0542 (6)	
C19	0.8037 (2)	0.17294 (14)	0.8276 (3)	0.0675 (8)	
H19A	0.8055	0.1899	0.9226	0.081*	
C20	0.7439 (2)	0.20858 (16)	0.7028 (4)	0.0774 (8)	
H20A	0.7061	0.2490	0.7133	0.093*	
C21	0.7405 (2)	0.18413 (16)	0.5622 (4)	0.0748 (8)	
H21A	0.6994	0.2078	0.4770	0.090*	
C22	0.7968 (3)	0.12537 (15)	0.5465 (3)	0.0729 (8)	
H22A	0.7943	0.1095	0.4505	0.088*	
C23	0.9249 (2)	0.07561 (13)	0.9551 (3)	0.0566 (7)	
C24	0.9348 (3)	0.10499 (16)	1.1066 (3)	0.0816 (9)	
H24A	1.0123	0.1058	1.1639	0.122*	
H24B	0.8931	0.0769	1.1578	0.122*	
H24C	0.9055	0.1513	1.0963	0.122*	
N1	0.53908 (15)	0.35937 (9)	0.4040 (2)	0.0459 (5)	
N2	0.47974 (15)	0.30681 (9)	0.3170 (2)	0.0471 (5)	
H2A	0.4611	0.3086	0.2200	0.057*	
N3	0.97077 (17)	0.01712 (11)	0.9343 (2)	0.0608 (6)	
O1	0.61634 (17)	0.40377 (9)	0.67081 (19)	0.0729 (6)	
H01A	0.5821	0.3756	0.5844	0.107 (11)*	
O2	0.47137 (14)	0.25029 (7)	0.52807 (18)	0.0522 (4)	
O3	0.33937 (14)	0.14856 (7)	0.36327 (18)	0.0517 (4)	
O4	0.91241 (19)	0.03129 (10)	0.6491 (2)	0.0892 (7)	
H04A	0.9261	0.0087	0.7578	0.175 (18)*	

### Atomic displacement parameters (Å^2^)

**Table:**

	*U*^11^	*U*^22^	*U*^33^	*U*^12^	*U*^13^	*U*^23^
C1	0.0671 (16)	0.0455 (13)	0.0432 (15)	0.0023 (12)	0.0097 (12)	−0.0008 (11)
C2	0.0467 (13)	0.0408 (12)	0.0417 (14)	0.0041 (10)	0.0097 (11)	0.0026 (10)
C3	0.0655 (16)	0.0484 (14)	0.0511 (16)	0.0014 (13)	0.0115 (13)	0.0074 (12)
C4	0.0721 (18)	0.0443 (14)	0.077 (2)	−0.0063 (13)	0.0051 (15)	0.0030 (14)
C5	0.082 (2)	0.0483 (15)	0.067 (2)	−0.0021 (14)	−0.0082 (16)	−0.0069 (13)
C6	0.095 (2)	0.0567 (16)	0.0462 (17)	−0.0006 (15)	−0.0006 (15)	−0.0079 (12)
C7	0.0485 (13)	0.0463 (12)	0.0365 (13)	0.0032 (11)	0.0135 (10)	0.0031 (10)
C8	0.0501 (13)	0.0475 (13)	0.0368 (14)	−0.0013 (11)	0.0184 (11)	−0.0025 (10)
C9	0.0664 (15)	0.0532 (14)	0.0450 (15)	−0.0109 (12)	0.0257 (12)	−0.0062 (11)
C10	0.0502 (14)	0.0469 (13)	0.0371 (13)	−0.0058 (11)	0.0099 (11)	−0.0008 (10)
C11	0.0601 (16)	0.0634 (15)	0.0513 (16)	−0.0061 (13)	0.0122 (13)	0.0148 (12)
C12	0.0552 (17)	0.103 (2)	0.061 (2)	−0.0151 (17)	0.0167 (14)	0.0130 (16)
C13	0.0546 (17)	0.102 (2)	0.081 (2)	0.0014 (18)	0.0205 (16)	−0.0114 (19)
C14	0.0607 (18)	0.0661 (18)	0.111 (3)	0.0056 (15)	0.0202 (18)	−0.0032 (17)
C15	0.0617 (17)	0.0495 (15)	0.085 (2)	−0.0031 (13)	0.0225 (15)	0.0097 (13)
C16	0.0799 (18)	0.0588 (15)	0.0392 (15)	−0.0051 (14)	0.0122 (13)	0.0047 (11)
C17	0.0699 (17)	0.0546 (15)	0.0647 (19)	−0.0133 (14)	0.0217 (15)	−0.0017 (14)
C18	0.0477 (14)	0.0530 (14)	0.0611 (18)	−0.0111 (12)	0.0138 (12)	−0.0037 (12)
C19	0.0584 (17)	0.0710 (17)	0.070 (2)	0.0003 (14)	0.0131 (15)	−0.0035 (15)
C20	0.0686 (19)	0.0786 (19)	0.083 (2)	0.0091 (16)	0.0178 (17)	0.0099 (18)
C21	0.0653 (18)	0.081 (2)	0.074 (2)	−0.0061 (16)	0.0125 (16)	0.0181 (17)
C22	0.082 (2)	0.0755 (19)	0.060 (2)	−0.0186 (17)	0.0184 (16)	0.0027 (15)
C23	0.0489 (14)	0.0579 (15)	0.0606 (18)	−0.0113 (12)	0.0111 (13)	−0.0047 (13)
C24	0.092 (2)	0.085 (2)	0.060 (2)	0.0082 (17)	0.0077 (16)	−0.0106 (16)
N1	0.0550 (12)	0.0468 (11)	0.0351 (11)	−0.0057 (9)	0.0112 (9)	−0.0037 (9)
N2	0.0601 (12)	0.0511 (11)	0.0301 (11)	−0.0107 (10)	0.0125 (9)	−0.0029 (8)
N3	0.0591 (13)	0.0617 (14)	0.0585 (15)	−0.0062 (11)	0.0112 (11)	0.0014 (10)
O1	0.1084 (15)	0.0686 (11)	0.0381 (11)	−0.0196 (11)	0.0143 (10)	0.0011 (9)
O2	0.0677 (11)	0.0575 (10)	0.0346 (10)	−0.0049 (8)	0.0193 (8)	−0.0016 (7)
O3	0.0626 (10)	0.0432 (8)	0.0565 (11)	−0.0034 (8)	0.0284 (9)	0.0022 (7)
O4	0.1350 (19)	0.0682 (12)	0.0699 (15)	0.0083 (13)	0.0374 (13)	−0.0032 (11)

### Geometric parameters (Å, °)

**Table:**

C1—O1	1.354 (3)	C14—C15	1.379 (4)
C1—C6	1.385 (3)	C14—H14A	0.9300
C1—C2	1.404 (3)	C15—H15A	0.9300
C2—C3	1.393 (3)	C16—H16A	0.9600
C2—C7	1.472 (3)	C16—H16B	0.9600
C3—C4	1.375 (3)	C16—H16C	0.9600
C3—H3A	0.9300	C17—O4	1.351 (3)
C4—C5	1.364 (4)	C17—C22	1.391 (4)
C4—H4A	0.9300	C17—C18	1.402 (4)
C5—C6	1.368 (4)	C18—C19	1.394 (3)
C5—H5A	0.9300	C18—C23	1.471 (3)
C6—H6A	0.9300	C19—C20	1.370 (4)
C7—N1	1.289 (3)	C19—H19A	0.9300
C7—C16	1.489 (3)	C20—C21	1.369 (4)
C8—O2	1.219 (3)	C20—H20A	0.9300
C8—N2	1.351 (3)	C21—C22	1.362 (4)
C8—C9	1.511 (3)	C21—H21A	0.9300
C9—O3	1.408 (3)	C22—H22A	0.9300
C9—H9A	0.9700	C23—N3	1.303 (3)
C9—H9B	0.9700	C23—C24	1.481 (4)
C10—C15	1.365 (3)	C24—H24A	0.9600
C10—C11	1.373 (3)	C24—H24B	0.9600
C10—O3	1.376 (3)	C24—H24C	0.9600
C11—C12	1.376 (4)	N1—N2	1.375 (2)
C11—H11A	0.9300	N2—H2A	0.8600
C12—C13	1.364 (4)	N3—N3^i^	1.394 (4)
C12—H12A	0.9300	O1—H01A	0.9610
C13—C14	1.364 (4)	O4—H04A	1.0635
C13—H13A	0.9300		
			
O1—C1—C6	117.0 (2)	C10—C15—C14	119.4 (3)
O1—C1—C2	123.1 (2)	C10—C15—H15A	120.3
C6—C1—C2	119.9 (2)	C14—C15—H15A	120.3
C3—C2—C1	117.5 (2)	C7—C16—H16A	109.5
C3—C2—C7	120.4 (2)	C7—C16—H16B	109.5
C1—C2—C7	122.0 (2)	H16A—C16—H16B	109.5
C4—C3—C2	121.8 (2)	C7—C16—H16C	109.5
C4—C3—H3A	119.1	H16A—C16—H16C	109.5
C2—C3—H3A	119.1	H16B—C16—H16C	109.5
C5—C4—C3	119.6 (3)	O4—C17—C22	117.6 (3)
C5—C4—H4A	120.2	O4—C17—C18	122.2 (3)
C3—C4—H4A	120.2	C22—C17—C18	120.2 (3)
C4—C5—C6	120.5 (2)	C19—C18—C17	116.8 (2)
C4—C5—H5A	119.7	C19—C18—C23	120.7 (2)
C6—C5—H5A	119.7	C17—C18—C23	122.5 (2)
C5—C6—C1	120.7 (3)	C20—C19—C18	122.6 (3)
C5—C6—H6A	119.7	C20—C19—H19A	118.7
C1—C6—H6A	119.7	C18—C19—H19A	118.7
N1—C7—C2	114.8 (2)	C21—C20—C19	119.3 (3)
N1—C7—C16	124.6 (2)	C21—C20—H20A	120.3
C2—C7—C16	120.62 (19)	C19—C20—H20A	120.3
O2—C8—N2	123.0 (2)	C22—C21—C20	120.5 (3)
O2—C8—C9	122.8 (2)	C22—C21—H21A	119.8
N2—C8—C9	114.2 (2)	C20—C21—H21A	119.8
O3—C9—C8	111.74 (19)	C21—C22—C17	120.7 (3)
O3—C9—H9A	109.3	C21—C22—H22A	119.7
C8—C9—H9A	109.3	C17—C22—H22A	119.7
O3—C9—H9B	109.3	N3—C23—C18	116.1 (2)
C8—C9—H9B	109.3	N3—C23—C24	123.1 (2)
H9A—C9—H9B	107.9	C18—C23—C24	120.8 (2)
C15—C10—C11	120.0 (2)	C23—C24—H24A	109.5
C15—C10—O3	125.1 (2)	C23—C24—H24B	109.5
C11—C10—O3	114.9 (2)	H24A—C24—H24B	109.5
C10—C11—C12	119.9 (2)	C23—C24—H24C	109.5
C10—C11—H11A	120.0	H24A—C24—H24C	109.5
C12—C11—H11A	120.0	H24B—C24—H24C	109.5
C13—C12—C11	120.3 (3)	C7—N1—N2	120.49 (19)
C13—C12—H12A	119.9	C8—N2—N1	116.77 (19)
C11—C12—H12A	119.9	C8—N2—H2A	121.6
C14—C13—C12	119.5 (3)	N1—N2—H2A	121.6
C14—C13—H13A	120.3	C23—N3—N3^i^	115.2 (3)
C12—C13—H13A	120.3	C1—O1—H01A	101.2
C13—C14—C15	120.9 (3)	C10—O3—C9	117.65 (17)
C13—C14—H14A	119.6	C17—O4—H04A	98.1
C15—C14—H14A	119.6		
			
O1—C1—C2—C3	−178.5 (2)	O4—C17—C18—C19	179.1 (2)
C6—C1—C2—C3	0.8 (3)	C22—C17—C18—C19	−0.8 (4)
O1—C1—C2—C7	0.8 (4)	O4—C17—C18—C23	−0.4 (4)
C6—C1—C2—C7	−179.9 (2)	C22—C17—C18—C23	179.7 (2)
C1—C2—C3—C4	0.2 (4)	C17—C18—C19—C20	0.6 (4)
C7—C2—C3—C4	−179.1 (2)	C23—C18—C19—C20	−179.8 (2)
C2—C3—C4—C5	−0.9 (4)	C18—C19—C20—C21	0.1 (4)
C3—C4—C5—C6	0.6 (4)	C19—C20—C21—C22	−0.7 (4)
C4—C5—C6—C1	0.4 (4)	C20—C21—C22—C17	0.5 (4)
O1—C1—C6—C5	178.3 (3)	O4—C17—C22—C21	−179.7 (3)
C2—C1—C6—C5	−1.1 (4)	C18—C17—C22—C21	0.3 (4)
C3—C2—C7—N1	171.5 (2)	C19—C18—C23—N3	175.8 (2)
C1—C2—C7—N1	−7.7 (3)	C17—C18—C23—N3	−4.7 (3)
C3—C2—C7—C16	−7.9 (3)	C19—C18—C23—C24	−4.6 (4)
C1—C2—C7—C16	172.8 (2)	C17—C18—C23—C24	174.9 (2)
O2—C8—C9—O3	18.8 (3)	C2—C7—N1—N2	−178.66 (18)
N2—C8—C9—O3	−161.82 (19)	C16—C7—N1—N2	0.7 (3)
C15—C10—C11—C12	−0.8 (4)	O2—C8—N2—N1	4.7 (3)
O3—C10—C11—C12	−179.5 (2)	C9—C8—N2—N1	−174.67 (19)
C10—C11—C12—C13	0.3 (4)	C7—N1—N2—C8	−175.6 (2)
C11—C12—C13—C14	0.2 (5)	C18—C23—N3—N3^i^	−179.5 (2)
C12—C13—C14—C15	−0.3 (5)	C24—C23—N3—N3^i^	0.9 (4)
C11—C10—C15—C14	0.7 (4)	C15—C10—O3—C9	−0.9 (3)
O3—C10—C15—C14	179.3 (2)	C11—C10—O3—C9	177.8 (2)
C13—C14—C15—C10	−0.2 (5)	C8—C9—O3—C10	72.5 (3)

Symmetry codes: (i) −*x*+2, −*y*, −*z*+2.

### Hydrogen-bond geometry (Å, °)

**Table:**

*D*—H···*A*	*D*—H	H···*A*	*D*···*A*	*D*—H···*A*
N2—H2A···O2^ii^	0.86	2.14	2.860 (3)	141
O1—H01A···N1	0.96	1.63	2.530 (3)	154
O4—H04A···N3	1.06	1.58	2.542 (3)	148

Symmetry codes: (ii) *x*, −*y*+1/2, *z*−1/2.
